# Controllable continuous sub-tenon drug delivery of dexamethasone disodium phosphate to ocular posterior segment in rabbit

**DOI:** 10.1080/10717544.2016.1264498

**Published:** 2017-02-06

**Authors:** Xuetao Huang, Shaogang Liu, Yezhen Yang, Yiqin Duan, Ding Lin

**Affiliations:** 1 Department of Ophthalmology, Changsha Aier Hospital, Aier School of Ophthalmology, Central South University, Changsha, China, and; 2 Advanced Research Center, Central South University, Changsha, China

**Keywords:** Dexamethasone, sub-tenon drug delivery, posterior segment, rabbit eye, topical administration, pharmacokinetics

## Abstract

Corticosteroids have been used for treatment of posterior segment eye diseases, but the delivery of drug to the posterior segments is still a problem to resolve. In our study, we explore the feasibility of Sub-tenon’s Controllable Continuous Drug Delivery to ocular posterior segment. Controllable continuous sub-tenon drug delivery (CCSDD) system, intravenous injections (IV) and sub-conjunctival injections (SC) were used to deliver dexamethasone disodium phosphate (DEXP) in rabbits, the dexamethasone concentration was measured in the ocular posterior segment tissue by Shimadzu LC-MS 2010 system at different time points in 24 h after first dose injection. Levels of dexamethasone were significantly higher at 12, 24 h in CCSDD than two other approaches, and at 3, 6 h in CCSDD than IV in vitreous body (*p* < 0.01); at 6, 12, 24 h in CCSDD than two other approaches, and at 1, 3 h in CCSDD than IV in retinal/choroidal compound (*p* < 0.01); at 3, 6, 12, 24 h in CCSDD than two other approaches, and at 1 h in CCSDD than IV in sclera (*p* < 0.05). The AUC_0–24_ in CCSDD group is higher than two other groups in all ocular posterior segment tissue. Our results demonstrated that dexamethasone concentration could be sustained moderately higher in the posterior segment by CCSDD than SC and IV, indicating that CCSDD might be a therapeutic alternative to treat a variety of intractable posterior segment diseases.

## Introduction

Corticosteroids have been used for treatment of posterior segment eye diseases (Lowder et al., 2011; Chennamaneni et al., 2013; Calvo et al., 2015; Tsang et al., 2016) such as uveitis, diabetic retinopathy and macular degeneration which can lead to irreversible visual impairments (Hettinga et al., 2014; Borooah et al., 2015; Zatic et al., 2015). Inflammation of ocular posterior segment is often difficult to treat because of poor tissue permeability (Bourges et al., 2003; Bansal et al., 2016; Lajunen et al., 2016). Delivery of drug to the posterior segments is of great concern but also challenging due to the anatomical and physiological barriers of the eye and short drug duration.

Steroids used to treat posterior segment diseases are typically administered in four ways such as topical, systemic, intravitreal (IVT) and periocular routes (Kang-Mieler et al., 2014). The topical route is well tolerated (Hennessy et al., 2010; Quek et al., 2011), but inefficient in delivering therapeutic drug levels (Chen et al., 2012; Liu et al., 2016; Thakur et al., 2016), owing to rapid drainage through the nasolacrimal ducts, systemic absorption, low permeability of the corneal (Abdul et al., 2016) and conjunctival epithelium, and the blood–aqueous barrier. Systemic administrations of corticosteroid show low bioavailability (Thakur et al., 2016) and significant systemic side effects, it require large dosage for the effective therapeutic concentration in the eye (Andonova, 2016), nonspecific accumulation of drugs in other organs, the blood–retina barrier, and not well tolerated by all patients (Peptu et al., 2015). Intravitreal injections or implants can deliver drugs to the posterior segments effectively and it can reach the targeted tissue directly with minimized systemic side effects. But it is the most invasive, and can lead to numerous significant local complications such as retinal drug toxicity, retinal detachment, vitreous and retinal hemorrhage, floaters, endophthalmitis, lens injury, and elevated intraocular pressure, which will affect the vision of patients (Huang et al., 2016). Consequently, the ideal routes of drug delivery to the posterior segment are from the eye of periocular (Hsu, 2007), which is considered to be minimal painful and direct penetration pathway (Nagai et al., 2014; Huang et al., 2016). It included the retrobulbar, peribulbar, sub-tenon and subconjunctival (SC) routes. Both the SC and the sub-tenon routes are widely used in research of transscleral drug delivery as their neighboring layer is the sclera. With SC injection, the formulation is placed beneath the conjunctival membrane that covers the sclera. This enables the drugs to bypass the conjunctiva–cornea barrier, giving direct access to the transscleral route. Sub-tenon injection involves the placement of a formulation between the sclera and Tenon’s capsule, an avascular membrane. Therefore, the contact time between the administered drug and the sclera is prolonged. Consequently, the sub-tenon route is considered to be one of the most promising routes for targeting the posterior segment (Ghate et al., 2007). But it is also restricted for the short duration in most of the drugs currently used. Sub-tenon drug delivery system is a better choice for ophthalmologists, because it is not only for the greater bioavailability but also the higher processing safety. The sub-tenon’s route is regarded as the most effective one for it can lay the agents proximal to the sclera (Thrimawithana et al., 2011).

Researches showed that sub-tenon capsule sustained release implants can release drugs and keep certain vitreous, retinal, and choroidal drug concentration, but it is in a lower level due to the limited total drug dosage. The aim of our study was to evaluate the feasibility of controllable continuous sub-tenon drug delivery (CCSDD) to ocular posterior segment in rabbit. To investigate whether the CCSDD system can release drugs such as the dexamethasone disodium phosphate (DEXP) and keep a higher level of corticosteroid in the posterior segment *in vivo*, and evaluate its distribution in the ocular posterior tissues.

## Methods and materials

### Animals

All experiments were carried out in accordance with the statement of the Association for Research in Vision and Ophthalmology (ARVO) for the Use of Animals in Ophthalmic and Vision Research. The studies were approved by the Animal Ethics Committee of the Third Xiangya Hospital of Central South University. Male and female New Zealand albino rabbits (about four months old and weighing 2.0–2.5 kg) were acquired from the Third Xiangya Hospital of Central South University (Changsha, China). All rabbits were examined thoroughly, and no ocular disease, then were randomly allocated to different groups for subsequent ocular PK studies. Animals were anesthetized by intramuscularly injecting Xylazine Hydrochloride Injection (2 ml:0.2 g, Huamu Animal Health Products Co., Jilin, 0.1–0.2 ml/kg) and dropped Oxybuprocaine Hydrochloride Eye Drops (20 ml:80 mg, Sa) three times, interval 2 min per time.

### Drug administration and sample collection

**Figure 1.**
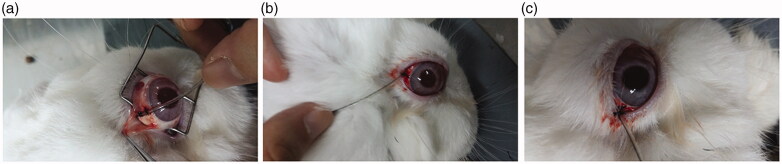
The picture of sub-tenon embedded tubes in rabbit: (a) the tube sutured on sclera, (b) Sutured on the conjunctive edge, and (c) The tube sutured in the eyelid.

**Chart 1.**
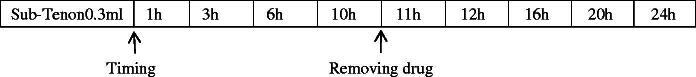
The illustration of the time in CCSDD group.

Rabbits were randomly divided into three groups and six rabbits for each group per time point. Intravenous injection (IV, 1 mg/kg dexamethasone), SC injection (5 mg/ml dexamethasone, 0.3 ml) and CCSDD system (CCSDD 0.3 ml initial doses, 5 mg/ml dexamethasone, 0.1 ml/h sustained pump) were performed. After the last instillation in IV group (1 h, 3 h, 6 h, 12 h, 24 h) and SC injection group (1 h, 2 h, 3 h, 4 h, 6 h, 8 h, 12 h, 24 h), respectively, the ocular tissue were collected. In the CCSDD group, after placing embedded tubes in sub-tenon (Figure 1), 0.3 ml 5 mg/ml dexamethasone trickled into it and starting from that time, the pump drugs at the rate of 0.1 ml/h to the scleral surface for 10 h (Chart 1), and collects plasma and ocular tissue in 1 h, 3 h, 6 h, 10 h, 11 h, 12 h, 16 h, 20 h, 24 h. Animals were euthanized by 3 ml lidocaine (3 ml/rabbit) and 3 ml air intravenously, immediately after death, ocular tissues were collected. Vitreous, choroid/retina and sclera were dissected and weighted as the solid tissues, the samples were stored at −20 °C and then processed for analysis.

### Drug assay

We determined the levels of dexamethasone in the rabbit samples by Shimadzu LC-MS 2010 system. Frozen rabbit samples were thawed at ambient temperature, the solid samples were fixed with 400 ml 0.9% normal saline (NS) and homogenized. A 100 μl aliquot of Triamcinolone diacetate (I.S. 200 ng/ml) standard solution was added to 100 μl of each fluid sample and 400 μl 0.9% NS or homogenized samples. After a thorough vortex mixing for 30 s, the mixture was extracted with 1 ml n-hexane and acetate ester (1:1, v/v) for 60 min, the mixture was centrifuged at 14 000 rpm for 5 min. The organic layer was removed and evaporated under a gentle stream nitrogen gas at 45 °C until it was completely dry. The dried residue was dissolved with 50 μl mobile phase. After centrifugation, 5 μl of the clear supernatant was injected into the LC–MS system: LC-10AD VP low pressure gradient pump, CTO-10A VP column temperature oven, SCL-10AD VP system controller, and LC-MS chemstation. Separation was achieved on a Thermo Hypersil-Hypurity C18 column (150 mm × 2.1 mm, i.d., 5 μm, USA) at 40 °C. Compounds were eluted up to a total retention time of 4.5 min using an isocratic mobile phase consisting of 5 mM ammonium formate (pH 4.0)–methanol acetonitrile (30:5:65, v/v/v) at 0.22 ml/min, and the injection volume was 5 μl. The operating parameters of ESI–MS (electrospray ionization–mass spectrometry) were as follows: capillary voltage was 4.5 kV; nebulizer nitrogen gas flow-rate was 1.5 l/min; drying N_2_ flow was 10 l/min; drying gas temperature was 250 °C, the gas used was of high purity, and system control and data evaluation were carried out using LC-MS chemstation (Japan). The mass selective detector (MSD) was operated in the positive ionization mode with selected-ion monitoring (SIM) at 393.6 for dexamethasone (*m*/*z*) and *m*/*z* 479.6 for Triamcinolone diacetate. Quantitation was performed by a linear regression analysis of peak areas ratio from a standard curve containing seven standard points.

### Pharmacokinetic and statistical analysis

Dexamethasone concentrations were analyzed for pharmacokinetics in different tissues respectively, which are calculated using kinetica 5.1 (kinetica 5.1, Innaphase, Philadelphia, PA) software by a model Non Compartmental of Extravascular analysis. The following pharmacokinetic parameters were obtained: peak concentration (Cmax), time to peak concentration (Tmax), elimination half-life (*T*_1/2_), area under the concentration–time curve between 0 and 24 h (AUC_0–24_).

Statistical analyses were performed by SPSS 18.0 (SPSS Inc., Chicago, IL). Data of dexamethasone concentrations were expressed as mean ± standard deviation. Tissue concentrations were analyzed using one-way analysis of variance (ANOVA). If there were differences in three groups, we used Dunnett T3 to analyze two groups. All other results were descriptive measures. Statistical significance was accepted at a level of *p* < 0.05.

## Results

### Concentration of dexamethasone

**Figure 2.**
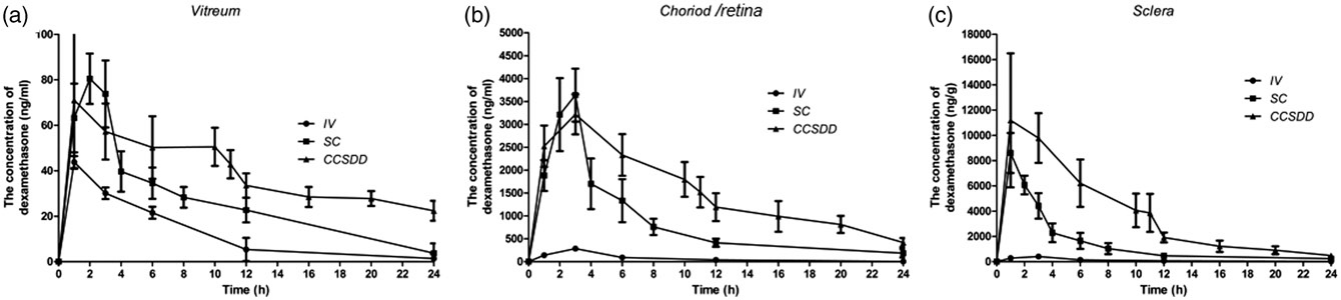
The mean levels of dexamethasone concentrations in the posterior segment tissue at different times in vitreous (a), choroid/retina compound (b), and sclera (c).

The mean levels of dexamethasone concentrations in the posterior segment tissue at different time intervals following IV, SC and CCSDD administration are shown in Table 1, and the results are presented in Figure 2.

**Table 1.** The dexamethasone concentrations in the posterior segment tissue following CCSDD, IV, SC.

	Vitreum	Choroidal/retinal compound	Sclera
CCSDD group			
**1 h**	71.05 ± 30.23	2522.46 ± 425.94	11192.22 ± 5302.51
**3 h**	57.23 ± 12.34	3291.35 ± 438.11	9785.20 ± 1964.12
**6 h**	50.23 ± 13.77	2331.09 ± 454.41	6215.17 ± 1871.83
10 h	50.51 ± 8.40	1796.19 ± 380.36	4053.03 ± 1330.47
11 h	42.84 ± 6.24	1518.52 ± 332.81	3857.47 ± 1508.05
**12 h**	33.52 ± 5.31	1191.62 ± 303.29	1913.73 ± 389.28
16 h	28.47 ± 4.42	988.97 ± 336.43	1215.48 ± 449.07
20 h	27.78 ± 3.26	812.90 ± 188.26	906.40 ± 312.84
**24 h**	22.40 ± 4.38	418.20 ± 97.81	484.52 ± 155.25
SC group			
**1 h**	63.19 ± 15.12	1881.83 ± 336.98^a^	8604.95 ± 1597.26
2 h	80.45 ± 11.10	3212.63 ± 796.76	6071.65 ± 737.76
**3 h**	73.77 ± 14.74	3638.80 ± 580.05	4419.68 ± 1006.87^a^
4 h	39.65 ± 8.87	1701.27 ± 551.90	2287.56 ± 742.66
**6 h**	34.53 ± 6.89	1333.65 ± 469.43^a^	1645.75 ± 631.69^a^
8 h	28.29 ± 4.56	760.86 ± 177.74	1031.78 ± 443.14
**12 h**	22.68 ± 5.40^a^	412.78 ± 88.89^a^	463.21 ± 136.14^a^
**24 h**	3.71 ± 4.31^a^	184.59 ± 94.86^a^	237.94 ± 139.25^a^
IV group			
**1 h**	43.78 ± 2.62	140.57 ± 34.56^a^	281.42 ± 60.13^a^
**3 h**	30.08 ± 2.55^a^	284.82 ± 23.14^a^	402.99 ± 96.49^a^
**6 h**	21.53 ± 2.66^a^	90.75 ± 12.08^a^	141.57 ± 31.46^a^
**12 h**	5.34 ± 5.11^a^	38.94 ± 13.00^a^	64.38 ± 11.83^a^
**24 h**	1.37 ± 2.14^a^	3.71 ± 4.50^a^	22.23 ± 7.44^a^

CCSDD group: the dexamethasone concentration in rabbit posterior segment at 1, 3, 6, 10, 11, 12, 16, 20 and 24 h after first dose of 0.3 ml (5 mg/ml) DEXP, and then continuous release of DEXP (5 mg/ml) at the rate of 0.1 ml/h for 10 h. SC group: the dexamethasone concentration at 1, 2, 3, 4, 6, 8, 12 and 24 h after subconjunctival injection of 0.3 ml (5 mg/ml) DEXP, IV group: the dexamethasone concentration at 1, 3, 6, 12 and 24 h after intravenous injection of 1 mg/kg DEXP in the rabbits. (Comparing the dexamethasone concentration at 1, 3, 6, 12 and 24 h between the three groups, ^a^denotes that compared with CCSDD group, the difference was statistically significant.). The bold values (1h, 3h, 6h, 12h, 24h) is the same time point in three group.

**Figure 3.**
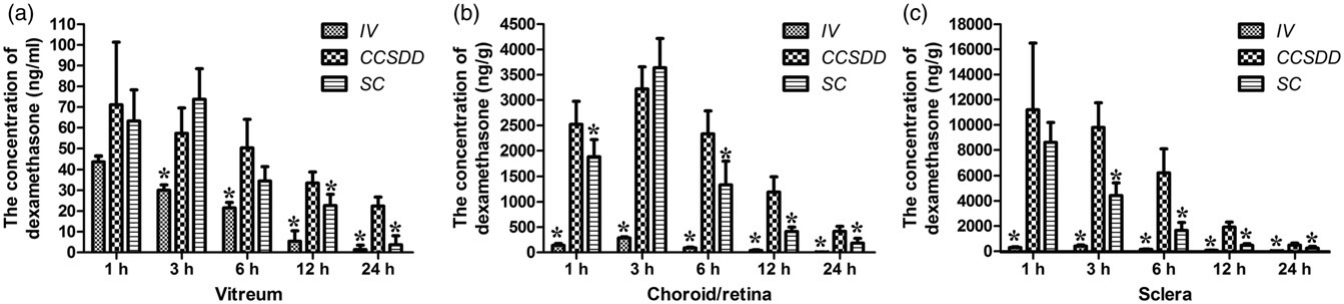
Levels of dexamethasone concentration in vitreous (a), choroid/retina compound (b), sclera (c). (*denotes that compared with CCSDD group, the difference was statistically significant).

Levels of dexamethasone concentration in the vitreous (Figure 3A) humor were significantly higher at 12 and 24 h (*n* = 6 per time per group, *p* < 0.05) and negligible at 1, 3, 6 h between CCSDD and SC (*n* = 6 per time per group, *p *= 0.917 at 1 h, *p *= 0.164 at 3 h, *p* = 0.105 at 6 h); and significantly higher at 3, 6, 12 and 24 h (*n* = 6 per time per group, *p* < 0.01) and negligible at 1 h between CCSDD and IV (*p *= 0.191).

Levels of dexamethasone concentration in the choroid/retinal compound (Figure 3B) were significantly higher at 1, 6, 12 and 24 h (*n* = 6 per time per group, *p* < 0.05) and negligible at 3 h between CCSDD and SC (*n* = 6 per time per group, *p *= 0.586); and significantly higher at 1, 3, 6, 12 and 24 h between CCSDD and IV (*p* < 0.01).

Levels of dexamethasone concentration in the sclera (Figure 3C) were significantly higher at 3, 6, 12 and 24 h (*p* < 0.05) and negligible at 1 h between CCSDD and SC (*n* = 6 per time per group, *p *= 0.612); and significantly higher at 1, 3, 6, 12 and 24 h between CCSDD and IV (*p* < 0.01).

### Pharmacokinetics

The pharmacokinetic parameters of dexamethasone in the rabbits' tissue are shown in Table 2.

**Table 2.** The pharmacokinetic parameters of dexamethasone in the rabbits' posterior segment tissue following CCSDD, IV, SC.

	Vitreum	Choroidal/retinal compound	Sclera
CCSDD group			
Cmax (ng/ml)	71.05	3219.35	11192.20
Tmax (h)	1	3	1
AUC_0–24_ (ng*h/ml)	1411.65	41400.70	93577.80
*T*_1/2_ (h)	14.32	7.65	4.89
SC group			
Cmax (ng/ml)	80.45	3638.80	8604.95
Tmax (h)	2	3	1
AUC_0–24_ (ng*h/ml)	628.84	22411.70	35582.30
*T*_1/2_ (h)	5.51	8.29	6.26
IV group			
Cmax (ng/ml)	43.78	284.82	402.99
Tmax (h)	1	3	3
AUC_0–24_ (ng*h/ml)	285.23	1571.71	2858.71
*T*_1/2_ (h)	4.52	3.85	6.88

Cmax: peak concentration; Tmax: time to peak concentration; AUC_0–24_: area under the concentration–time curve between 0 and 24 h; *T*_1/2_: elimination half-life.

Following the IV route, the maximum concentrations (Cmax) were 43.78 ng/ml, 284.82 ng/g and 402.99 ng/g in vitreous body, choroid/retinal compound and sclera, respectively. The time of maximum concentration (Tmax) was 1 h, 3 h, 3 h, respectively. The AUC_0–24_ were 285.23 ng·h/ml, 1571.71 ng·h/g and 2858.71 ng·h/g, respectively. The *T*_1/2_ were 4.52 h, 3.85 h, 6.88 h, respectively.

Following an SC administration, the peak average dexamethasone levels (Cmax) were 80.45 ng/ml, 3638.80 ng/g and 8604.95 ng/g, respectively, in vitreous body, choroid/retinal compound and sclera, and the Tmax were 2 h, 3 h, 1 h, respectively. The AUC_0–24_ were 628.84 ng·h/ml, 22411.70 ng·h/g and 35582.30 ng·h/g, respectively. The *T*_1/2_ were 5.51 h, 8.29 h and 6.26 h, respectively.

Following an CCSDD administration, the Cmax were 71.05 ng/ml, 3219.35 ng/g and 11192.20 ng/g in vitreous body, choroid/retinal compound and sclera, respectively, and the Tmax were 1 h, 3 h, 1 h, respectively. The AUC_0–24_ were 1411.65 ng·h/ml, 41400.70 ng·h/g and 93577.80 ng·h/g, respectively. The *T*_1/2_ were 14.32 h, 7.65 h, 4.89 h, respectively.

In IV and SC, it was the maximum dosage, and we observed that the AUC_0–24_ in CCSDD group is higher and the concentration of dexamethasone levels were not less than two other groups in all ocular posterior segment tissues.

## Discussion

Effectively administrating drugs to the posterior segment of the eye is very important for the treatment of the retinal, choroidal and vitreous diseases (Edelhauser et al., 2010). The aim of the present study was to develop a way of drug delivery that could release dexamethasone in a sustained and controllable manner at a given period and could be applied locally to the outer part of the sclera. For this purpose, the pump was selected to sustain release drug. Typical release profiles from pump was characterized by an initial push drug following the tube suturing the scleral surface, then by a controllable continuous release of dexamethasone at the rate of 0.1 ml/h in concentration of 5 mg/ml. In this report, we presented data showing the feasibility of delivering dexamethasone in a controllable and continuous mode by pump.

The sclera, a highly porous sponge-like tissue consisting of 68% water, is permeable to a series of hydrophilic compounds with various molecular weight compounds from 4 to 150 kDa (Janoria et al., 2007; Pescina et al., 2015). Iatrogenic perforation of the sclera at the injection site did not result in increased intraocular delivery, which indicated that lateral surface diffusion did not play a significant role in transscleral entry (Ambati et al., 2000), it demonstrates that sclera is porous indirectly. Human sclera facilitates diffusion due to its hypocellularity and large surface area, and its remarkable tolerance of foreign bodies overlying its surface makes it a long-term transscleral delivery device to be clinically feasible. The transscleral intraocular tissue distribution of corticosteroids was primarily driven by the drug solubility (Thakur et al., 2011). Scleral permeability depends on the molecular radius rather than the molecular lipophilicity (Ambati et al., 2000) and molecules of up to 70 000 Da can readily penetrate the sclera (Ambati et al., 2000). Subconjunctival injections increase the absorptive capacity of the sclera and lead to systemic absorption (Weijtens et al., 1997), the gradual delivery used in these experiments may permit more complete scleral absorption because the drug sclera contact time is greater than the lag time to steady state flux. Sub-tenon drug delivery deposited the therapeutic agents against the external surface of the sclera, which can avoid the risk of the intraocular drug delivery, make it safe and efficient to deliver the agents to posterior segment. So we selected the sub-tenon way to implant controllable continuous drug delivery tube.

An important aspect of continuous controllable-delivery system is that they provide continuous release, high concentrations of drug to achieve the desired pharmacological response. We sutured the tube to the sclera surface to make it close proximity to the sclera, increase drug sclera contact, thus improving scleral absorption, this can increase the bioavailability of the CCSDD, and the release time and rate can be adjusted according to the need. DEXP, a water soluble phosphate of dexamethasone, of which the molecular mass is 516.41 Da, can leak out from the tube and easily pass the sclera to the choroids. Dexamethasone has been used frequently in ocular posterior-segment treatments. The high potency and relatively acceptable toxicity of this compound, combined with its multifunctional roles as an anti-inflammatory, antiangiogenic and antipermeability agent, confirm that it is a candidate for various ocular drug-delivery platforms. Overall, the high concentrations of the dexamethasone in the posterior segments and the characteristic of adjustability of drug release rate and time, ensure that the CCSDD can act as an alternative choice to the posterior drug delivery.

We set the SC and IV of DEXP as control group for SC as it is the easiest and most popular method among periocular injection routes applied by the ophthalmologists (Hosseini et al., 2008; Hamdan et al., 2015; Liu et al., 2015) and IV is the basic method for most refractory ocular posterior segment disease. But we did not compare CCSDD and IVT, because IVT is invasive, and can lead to numerous significant local complications (Smith et al., 2014), meanwhile it cannot be repeated everyday. We observed that the level of dexamethasone in CCSDD group was greater than or equal to SC and IV at any time in all ocular posterior segment tissue. In CCSDD group, the levels of dexamethasone in posterior segment tissue at 24 h were roughly equal to the concentration levels in SC group at 12 h. Meanwhile, the levels of dexamethasone in posterior segment tissue at 24 h were almost disappearing in SC and IV. This demonstrated that the administration of CCSDD can act as an option for the refractory posterior segment diseases. AUC_0–24_ in CCSDD group is higher than other two groups. The dexamethasone exposure to vitreous humor in CCSDD group (AUC_0–24_=1411.65 ng·h/ml) was about five times as much as the AUC_0–24_ in IV group, 2.24 times in SC group. In choroid/retinal component (AUC_0–24_=41400.70 ng·h/g), it was about 26.34 times as much as in IV group, 1.85 times in SC group. In sclera (AUC_0–24_=93577.80 ng·h/g), it was 32.74 times as much as in IV group, 2.63 times in SC group.

We propose a novel sub-tenon approach for drug delivery via a sutured tube connecting to the scleral surfaces with a pump, we hypothesize that tube can infuse solutions containing soluble molecules, nanoparticles, and microparticles into the sclera in a minimally invasive manner. This would enable delivery of free drug or drug encapsulated within nanoparticles or microparticles for controlled release over time on the scleral surface. Drug could then diffuse from the sclera to neighboring choroidal and retinal tissues to treated the posterior segment diseases.

In SC group, the Cmax and Tmax in vitreous body were similar to those in previous study. When a depot corticosteroid preparation is injected subconjunctivally and side effect occurs, the remainder of the depot can be surgically removed (Huang et al., 2016), it is similar in CCSDD. Intrascleral drug delivery to the eye using hollow microneedles can infuse solutions into the sclera for minimally invasive delivery of soluble molecules, nanoparticles and microparticles (Jiang et al., 2009). But it is only experimented *in vitro*, and it may have the defect of perforated eye. Compared to the most popular IVT dexamethasone implant (Miserocchi et al., 2016), the CCSDD of the tube allowing the replenishment of the drug, facilitates for the need for a long time therapy. More importantly, although the CCSDD needs surgical implantation to the sub-Tenon’s sac, it does not mechanically break the normal structure of the retina, choroids and can be easily removed if severe complication occurs. As the IVT implantation needs surgical penetration of the sclera, choroids and retina, it can wander in the vitreous cavity and cause vitreous traction. What is more, if severe complication occurs, removal of the drug was not so easy for the ophthalmologists. Compared with controlled release formulations (Meng et al., 2014; Huang et al., 2016), CCSDD can reach a higher drug level in designed time and can control the rate of drug release.

At present, there are ultrasound-mediated transscleral deliveries (Suen et al., 2013), across the conjunctiva using iontophoresis (Eljarrat-Binstock & Domb, 2006) and transscleral iontophoresis administration (Eljarrat-Binstock et al., 2005,2008). While avoiding the complications of intraocular injection, these transscleral methods nonetheless involve hypodermic injection or implantation or the use of sophisticated electronic devices applied to the ocular surface. Though there was result suggesting that repeated transscleral iontophoresis drugs may be safe for treatment of ocular disorders (Patane et al., 2013), there was still not enough evidence to prove that iontophoresis and ultrasound may not cause any damage to the eye tissue. Meanwhile CCSDD delivers drug only through physical penetration, without any damage to the eye tissue, which can release drugs continuously at a controllable speed. However, there are also some limitations in our study. First, our data were observed only based on rabbits not humans, both species are different even similar. Rabbits’ eyes are smaller and have a thinner sclera, which facilitates drug penetrations into the choroid. They have a higher ocular flow rate and blood circulation rate, which may lead to a shorter half-life time of drug in rabbit ocular tissue. They have a much smaller body weight than humans. All of them can affect the ocular drug pharmacokinetic progress. Furthermore, this article only considered healthy rabbit’s eyes. Future work should investigate in diseased eyes (uveitis). Finally, it is only the preliminary study, the CCSDD system should be designed more simple and practical.

## Conclusions

In conclusion, the CCSDD system in this study could provide sustained release of DEXP to the posterior segment of the eye and it showed good bioavailability. It seems that the CCSDD can act as an alternative choice for the transscleral drug delivery to the posterior segment of the eye. It can release drugs precisely controlled. However, the CCSDD system is only the preliminary study, and should improve the comfort in the future for clinical using.
